# Comparing the effects of Bone^+^B® xenograft and InterOss® xenograft bone material on rabbit calvaria bone defect regeneration

**DOI:** 10.1002/cre2.875

**Published:** 2024-05-26

**Authors:** Afshin Yadegari Naini, Sepehr Kobravi, Aida Jafari, Mohammadhassan Lotfalizadeh, Narges Lotfalizadeh, Sareh Farhadi

**Affiliations:** ^1^ Department of Oral and Maxillofacial Surgery, Faculty of Dentistry (Khorasgan) Isfahan Azad University Isfahan Iran; ^2^ Department of Oral and Maxillofacial Surgery, Faculty of Dentistry, Tehran medical sciences Islamic Azad University Tehran Iran; ^3^ Department of Oral and Maxillofacial Radiology North Khorasan University of Medical Sciences Bojnurd Iran; ^4^ Department of Clinical Sciences, Faculty of Veterinary Medicine Ferdowsi University of Mashhad Mashhad Iran; ^5^ Department of Oral & Maxillofacial Pathology, Faculty of Dentistry, Tehran Medical Sciences Islamic Azad University Tehran Iran

**Keywords:** bone regeneration, calvarium, rabbit, xenograft

## Abstract

**Background:**

The bone regeneration therapy is often used in patients with inadequate bone support for implants, particularly following tooth extractions. Xenografts derived from animal tissues are effective bone reconstructive options that resist resorption and pose a low risk of transmitting disease. Therefore, these implants may be a good option for enhancing and stabilizing maxillary sinuses. The purpose of this study was to compare two xenografts, Bone^+^B® and InterOss®, for the reconstruction of rabbit calvaria defects.

**Methods and Materials:**

The study involved seven male New Zealand white rabbits. In the surgical procedure, 21 spots were created on both sides of the midline calvarium by creating three 8‐millimeter defects. A control group was used, as well as two treatment groups utilizing Bone^+^B® Grafts and InterOss® Grafts. After 3 months, the rabbits were euthanized, followed by pathological evaluation. Analysis of these samples focused on bone formation, xenograft remaining material, and inflammation levels, using Adobe Photoshop CS 8.0 and SPSS version 24.

**Results:**

With the application of Bone^+^B® graft, bone formation ranged from 32% to 45%, with a mean of 37.80% (±5.63), and the remaining material ranged from 28% to 37%, with a mean of 32.60% (±3.65). Using InterOss® grafts, bone formation was 61% to 75%, the mean was 65.83% (±4.75), and the remaining material was 9% to 18%, with a mean of 13.17% (±3.06). The bone formation in the control group ranged from 10% to 25%, with a mean of 17.17% (±6.11). InterOss® had lower inflammation levels than other groups, but the difference was not statistically significant (*p* > .05).

**Conclusion:**

InterOss® bone powder is the best option for maxillofacial surgery and bone reconstruction. This is due to more bone formation, less remaining material, and a lower inflammation level. Compared to the control group, Bone^+^B® improves healing and bone quality, thus making it an alternative to InterOss®.

## 1 INTRODUCTION

There is a common occurrence in which patients lack sufficient bone support to fully support implants when placed. Bone regeneration therapy is usually administered before or concurrently with implant insertion. Reconstruction and augmentation of skeletally damaged areas through bone grafting can be accomplished surgically (Raghoebar et al., 2001). One of the most common operative procedures requiring bone grafts in modern medicine is bone regeneration, especially in the jaw region due to bone loss following tooth extraction (Sakkas et al., 2017). Depending on the clinical condition of the patient, different treatment methods may be employed.

The first scenario involves mounting the graft components directly into the socket following tooth removal. During the second scenario, bone grafts are employed where teeth are unretained due to ridge resorption, making reconstruction necessary (Monje & Nart, 2022). These graft materials encourage bone regeneration by stimulating the body to produce new bone cells. The graft materials are substituted with newly formed bone tissue as part of the regenerative procedure (Titsinides et al., 2019). There are four main types of graft materials that are commonly employed in reconstructive surgery: autografts, xenografts, allografts, and alloplasts (Sheikh et al., 2017).

An autogenous bone graft can be obtained from various locations, namely the calvaria, ramus, or iliac crest of the patient (Öztürk et al., 2021). Since it contains cells with osteogenesis and regeneration capacity, it is considered the gold standard when it comes to bone grafting (Robinson et al., 2020). There are drawbacks to autogenous grafts, especially their unpredictable resorption and nonavailability of intraoral areas for donation (Zhang et al., 2023).

The second type of graft resource is xenograft, which is derived from animal tissues (Gashtasbi et al., 2020). The bone reconstruction techniques have been successful in preparing implants for implantation and enhancing the maxillary sinuses (Canellas et al., 2021). The resistance of xenografts to resorption is one of their positive characteristics (Capella‐Monsonís & Zeugolis, 2021). It is imperative to note, however, that xenografts seem to pose little risk of diseases being transmitted, specifically viruses and prions (Fishman, 2022).

Alloplasts provide another potential bone substitute material consisting of porous hydroxyapatite, cemented hydroxyapatite, beta‐tricalcium phosphate, apatite polymers, and beta‐tricalcium phosphate (Imaniyyah & Herda, 2022). As hydroxyapatite has long‐lasting properties, beta‐tricalcium phosphate has quick absorption characteristics (Imaniyyah & Herda, 2022).

Allografts are fabricated bone products obtained from deceased human donors that are helpful in promoting bone healing (Josephson & Kuehnert, 2022). Certain surgical procedures involving bone augmentation can be accomplished by implanting them rather than autogenous bone. Allografts are advantageous since they do not require a donor site and result in less trauma for the patient (Caccamese, 2023).

Bone^+^B® consists of a nonorganic bone matrix derived from bovine cancellous bone following a chemical and thermal treatment process that removes organic materials such as proteins, DNA, blood cells, and fat. However, hydroxyapatite retains its porous and crystalline structure. The porous nature of Bone^+^B® powder has been confirmed by scanning electron microscopy (SEM). Bone^+^B®'s porous structure promotes angiogenesis and new bone growth in periodontal, maxillofacial, and oral surgeries (Gashtasbi et al., 2020).

InterOss® bone powder is manufactured from Australian cancellous bovine bone hydroxyapatite, which is commonly available in the United States and other countries. It is one of the most widely used dental bone graft substitutes, and it is prepared by using both physical and chemical methods. In a low‐temperature annealing process with an extremely low heating rate, InterOss® nonorganic bone was reliably fabricated (Valencia‐Llano et al., 2022a). SEM images of InterOss® samples reveal that the material retains cancellous bone characteristics. It can be seen that the pieces are fragmented, with irregular edges, rough surfaces, and pores of different sizes. Furthermore, identifiable structures resembling bones, trabeculae, and medullary cavities can be observed (Valencia‐Llano et al., 2022b).

The availability of Bone^+^B® powder in the Iranian market and its lower cost make it an affordable alternative to foreign xenografts like InterOss®. However, it is unclear whether Bone^+^B® can compete with international brands in terms of bone regeneration. The purpose of this study is to compare the osteoconductive properties of two products, Bone^+^B® and InterOss®, to determine what material is most suitable for the reconstruction of bone defects. It may be possible to see which of the two materials, InterOss® or Bone^+^B®, demonstrates more advanced capabilities in bone regeneration and resorption. A comparison will also be made between their performance and the control group's spontaneous healing.

Since rabbit calvarium has a similar bone formation process to alveolar bone, and because rabbits have a high metabolic rate and mature within 6 months, rabbits were used in this study to assess the properties of the xenografts. Since rabbits' bone remodeling rates are three times greater than humans', initial bone healing responses can be assessed after 3 months (Schlund et al., 2022). Bone graft experiments can be successfully conducted on rabbit calvaria, according to research. Likewise, rabbit calvaria are formed by intramembranous bone processes, as are alveolar bones (Paknejad et al., 2015). Chang et al. examined the effects of calcium phosphate combined with cyanoacrylate by creating four 8‐mm defects in rabbit calvaria (Chang et al., 2011). Similar studies conducted by Lee et al. and Hussain et al. evaluated the effects of three types of allografts on rabbit defects with an 8‐mm diameter (Hussain et al., 2012; Lee et al., 2010). Studies conducted recently suggest that a defect of 8 mm in rabbit calvaria can be useful in structural evaluations of graft quality for bone reconstruction. This approach was employed to evaluate both xenografts in the present study as well.

This study is aimed at evaluating the bone regeneration abilities of Bone^+^B® and InterOss® xenografts in rabbits. A set of specific objectives has been set to reach this overall goal. To begin with, these objectives involve evaluating bone formation induced by Bone^+^B® xenografts and InterOss® xenografts in rabbit calvaria defects, with comparisons drawn between each graft material and the control group. Further, the study evaluates residual graft material in Bone^+^B® and InterOss® calvaria bone defects. In addition, the study will investigate and compare the level of inflammation associated with Bone^+^B® and InterOss® grafts applied to calvaria defects in rabbits, both in comparison to a control group and individually.

## 2 METHODS AND MATERIALS

### 2.1 Study animals

During this study, seven male New Zealand white rabbits weighing between 2.5 and 3 kg were examined. The New Zealand white rabbits had been provided 2 weeks before experimentation with a standard diet and unlimited access to water.

### 2.2 Surgical procedure

Animals were anesthetized with 2% xylazine (5 mg/kg) and 10% ketamine (40 mg/kg) via intramuscular injection. The rabbit's head was shaved, and the surgical site was disinfected with 10% povidone‐iodine solution. A 5‐min second disinfection of the surgical site followed. Through a direct incision of 8–7 cm along the middle line, an anterior–posterior incision was performed. Periosteal elevators were employed to retract the periosteum and subperiosteal tissues to expose the rabbit's parietal bone. Three 8‐millimeter defects were created on both sides of the midline calvarium using an 8‐millimeter internal diameter trephine irrigated sufficiently with saline.

Three equal‐sized cavities were drilled through the calvarium, resulting in 21 different spots arranged in random distributions. One control group and two treatment groups were present in this experiment. The meningeal membrane is susceptible to damage due to its thinness, making it essential to take precautions before surgery.

The defects were divided into three groups according to the type of bone grafts implanted. No material was placed in the defects in the Control Group, whereas in the Treatment Groups, Bone^+^B® Grafts (NovaTeb Pars) and InterOss® Grafts (SigmaGraft) were used to fill them.

**Figure 1:**
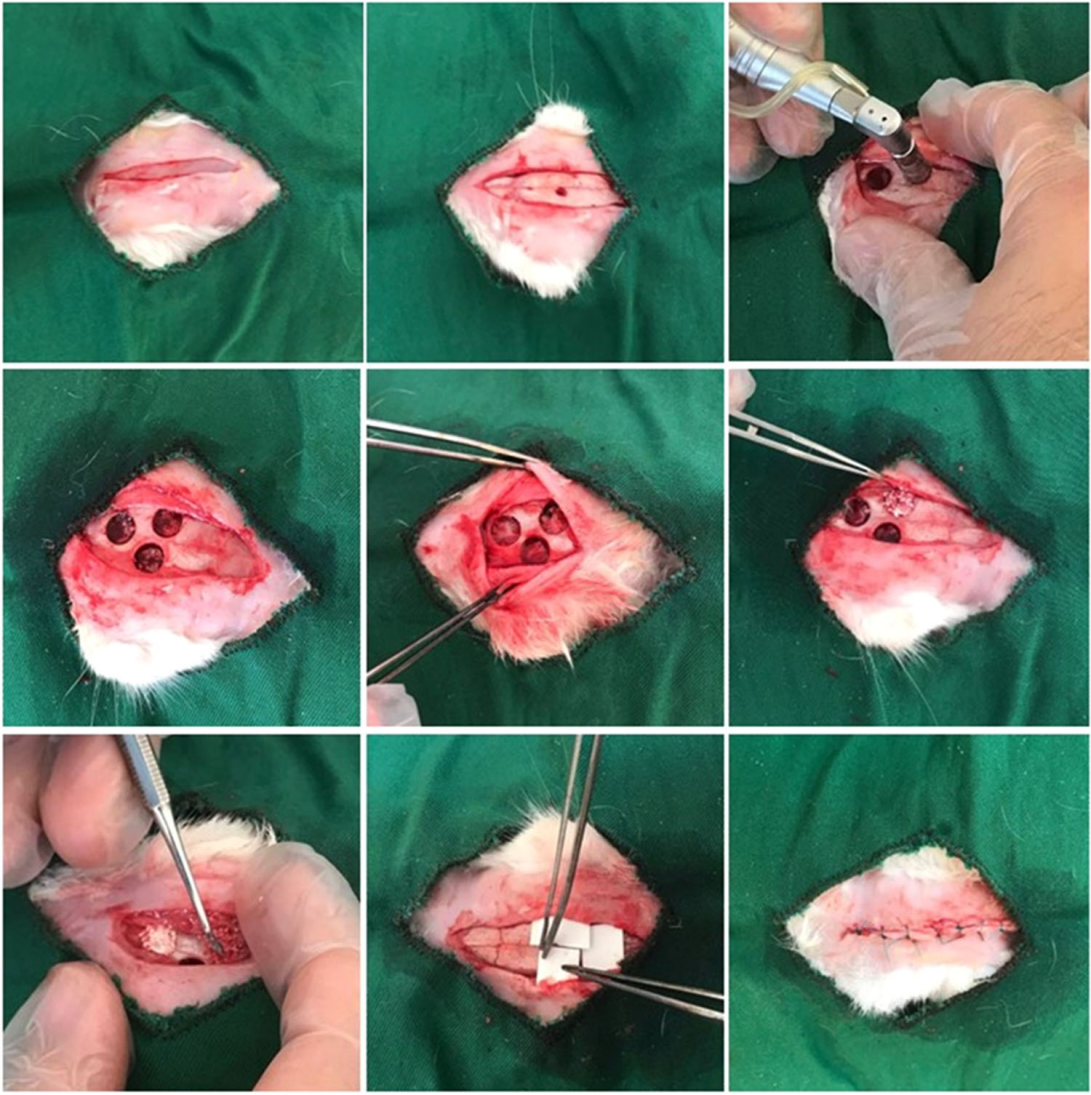
The surgical stages performed on the calvaria bone of a rabbit, creating three identical bone defects, and then implanting two types of Xenografts (InterOss® and Bone^+^B®) in two of them and leaving one empty as the control group.

A randomization principle was applied to assign the graft positions in the rabbits. Depending on the type of material used in the defects, the graft location in subsequent animals was altered to prevent errors. Over the filled defects in the rabbits, commercial TUTOPACH membranes (RTI Surgical) made from bovine pericardium were applied, thereby repositioning the periosteal flap. Sutures were placed in two layers to separate the periosteum and skin through absorbable Vicryl 4‐0 sutures (Figure 1).

Following surgery, the rabbits received subcutaneous injections of Enrofloxacin (10 mg/kg) and ketoprofen for 3 days. Veterinary examinations were conducted daily, and the animals were fed their normal diets.

### 2.3 Sampling

The study animals were euthanized intravenously with 100 mg/kg pentobarbital after 3 months of the surgery. After euthanasia, the calvarium was dissected with reciprocating saws using surgical scalpel blade No. 22 to separate the skin from the mucosa. An incision was made in the rabbit's occipital region to allow access to its parietal bone, and the defect areas were separated through saws. Afterward, samples were transferred to containers filled with 10% formalin. Within the pathology techniques, bone specimens were prepared.

**Figure 2:**
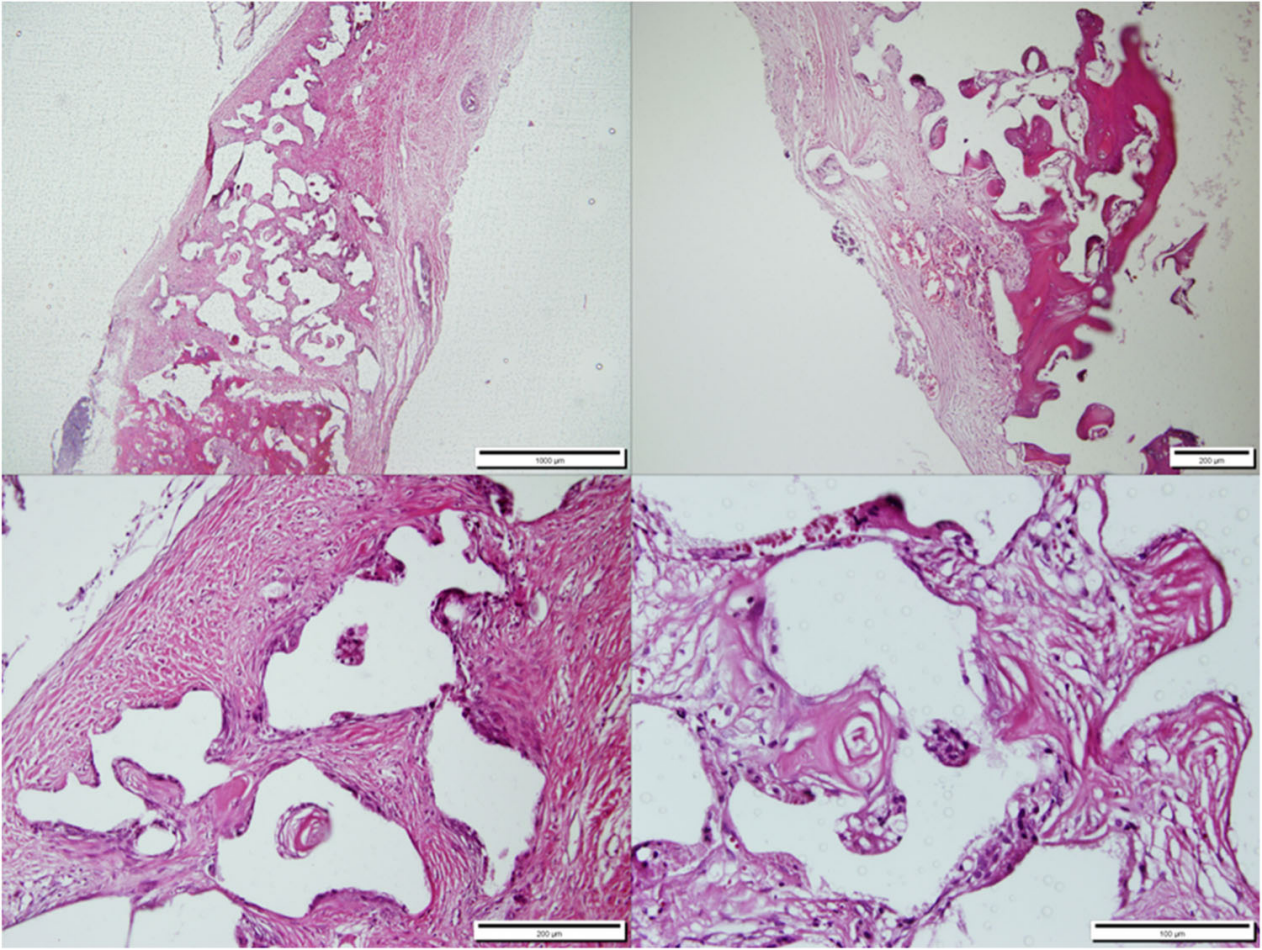
The histopathological sections obtained from the calvaria of the Bone^+^B® group rabbits, 3 months after defect creation, H&E staining, magnifications of 4×, 10×, 20×, and 40×.

**Figure 3:**
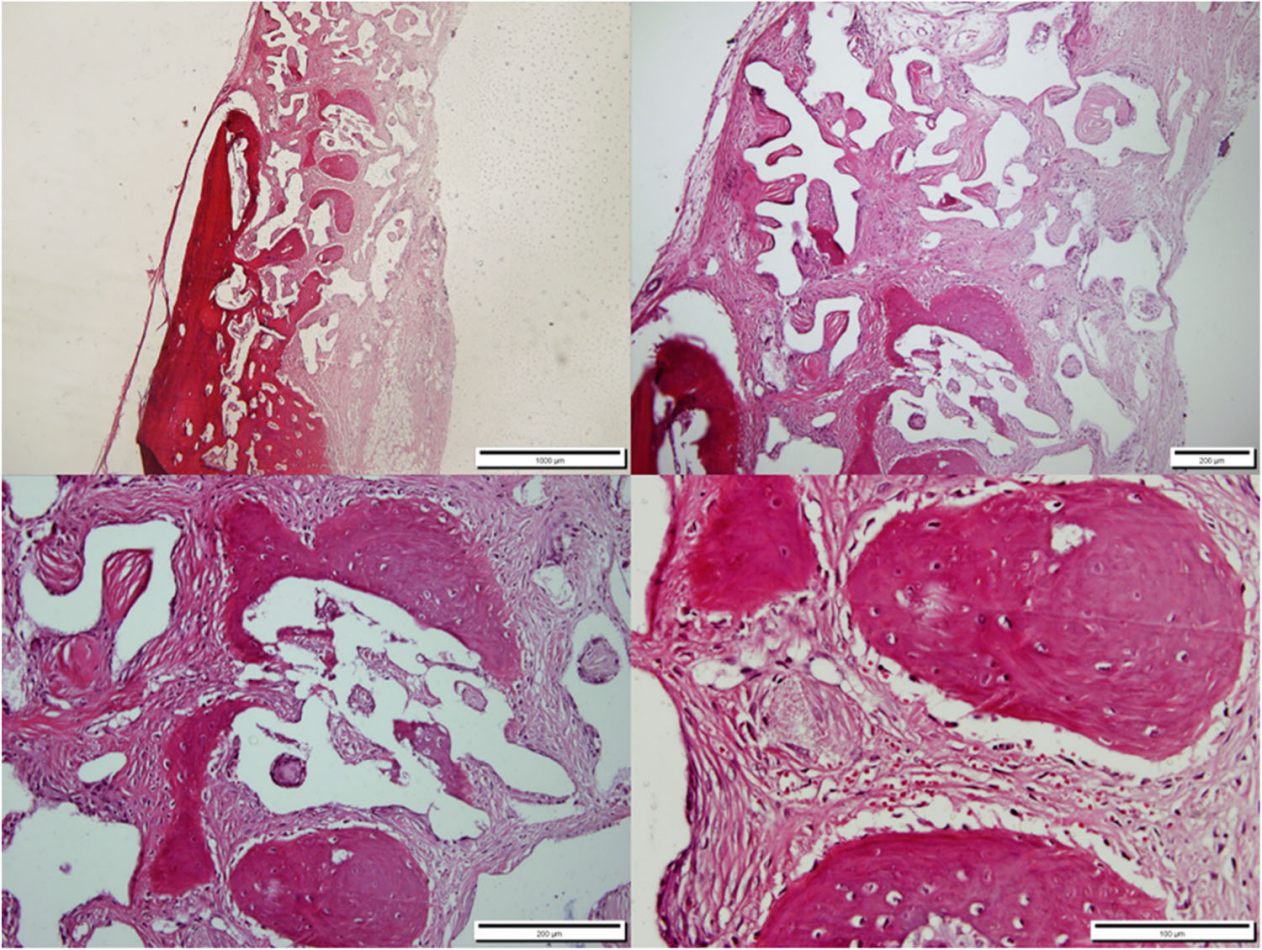
The histopathological sections obtained from the calvaria of the InterOss® group rabbits, 3 months after defect creation, H&E staining, magnifications of 4×, 10×, 20×, and 40×.

**Figure 4:**
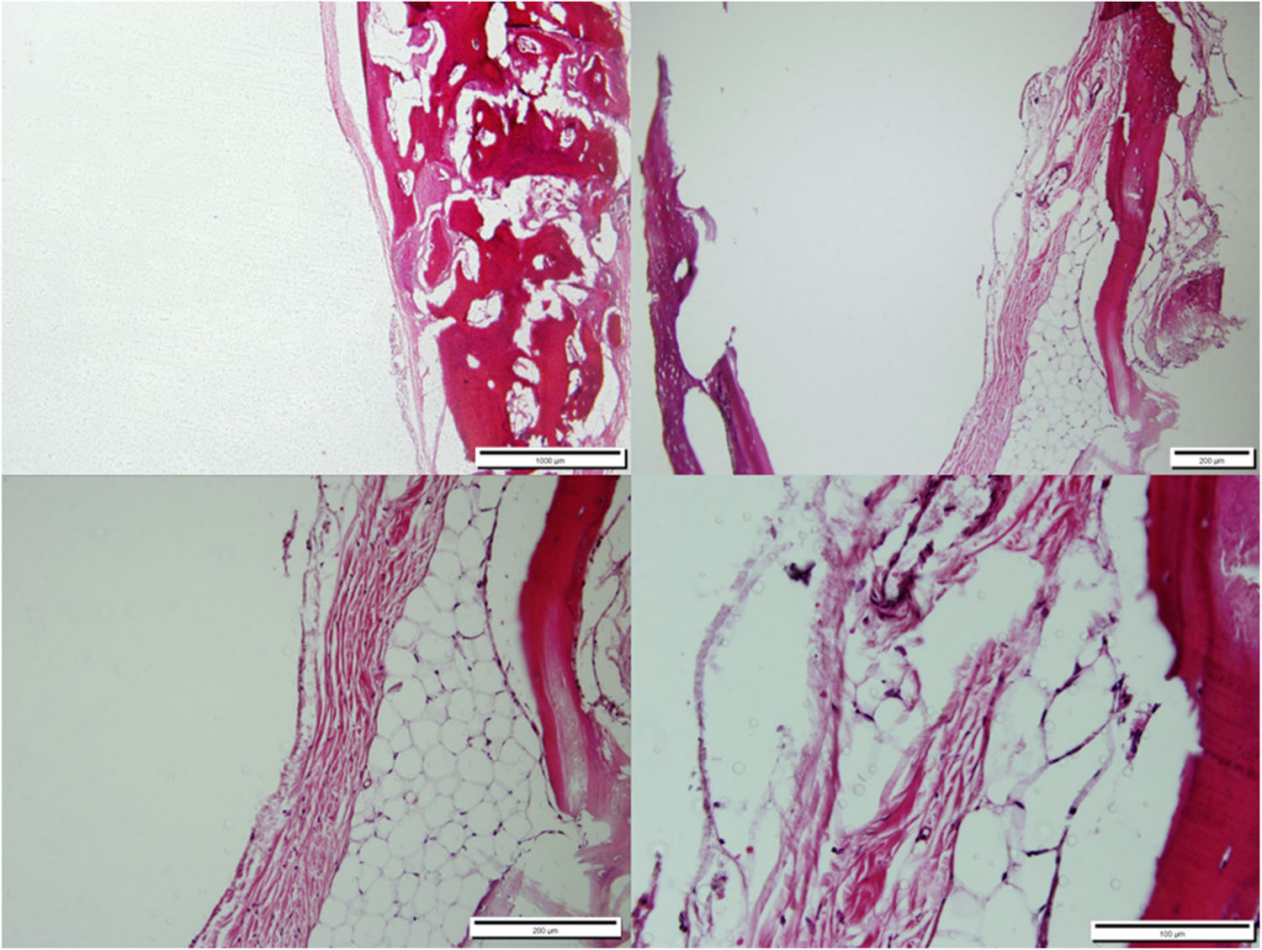
The histopathological sections obtained from the calvaria of the control group rabbits, 3 months after defect creation, H&E staining, magnifications of 4×, 10×, 20×, and 40×.

After keeping the specimens in 10% formalin solution for 5 days, the specimens were placed in 5% formic acid. Following dehydration in ethanol, the specimens were embedded in paraffin. As the decalcification process took up to 10 days, fresh acidic solution was applied daily, as well as daily evaluations of the decalcification progress. Hematoxylin and eosin (H&E) staining was performed on the sections (Figures 2, 3, 4).

Double‐blind evaluation by two pathologists was conducted on all slides produced by an automated system. Whenever there was disagreement between the two pathologists, the slides were assessed again by both and the results were recorded after discussion. Each sample was photographed using a computerized microscope at a magnification of 40×.

**Figure 5:**
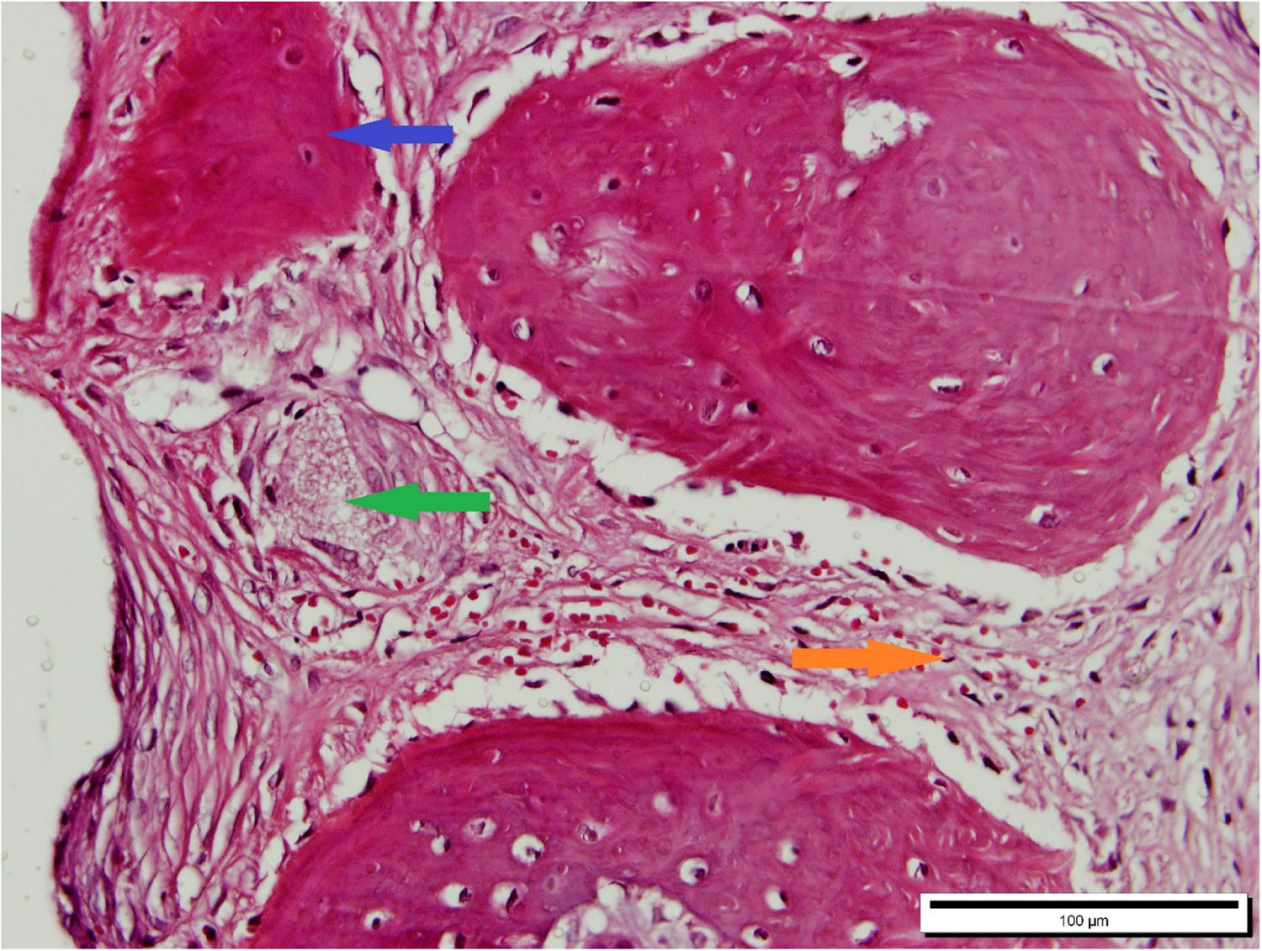
The microscopic view of rabbit calvaria after 3 months of grafting through a 40× objective lens. New bone formation is represented by the blue arrow, remaining xenograft material is indicated by the green arrow, and connective tissues are indicated by the orange arrow.

The level of Inflammation, bone formation percentage, and the amount of remaining Xenograft material were examined (Figure 5). A photomicroscope equipped with a BSW‐DP2 Olympus camera and connected to a computer was used for the evaluations, and the BSW‐DP2 software on the system was used for the assessments and measurements of these parameters.

The percentage of remaining materials and bone formation was calculated using Adobe Photoshop CS 8.0. Tests were conducted using SPSS version 24 with a 5% error margin. As a method of evaluating inflammation, a five‐grade system was applied. An inflammatory condition is categorized as grade 0 if there are no inflammatory cells; grade 1 is marked by mild, scattered inflammation; grade 2 is characterized by local and mild inflammation (5–10 inflammatory cells); and grade 3 is characterized by local and moderate

inflammation (10–50 inflammatory cells), and a grade 4 is defined by severe local inflammation (more than 50 inflammatory cells).

### 2.4 Statistical analysis

A Friedman test was used to compare quantitative variables, and the Cochran test was used for qualitative variables, and statistical analysis was carried out with SPSS version 24 with a significance level of 5%.

Two levels of analysis were conducted, one descriptive and one inferential. A descriptive level was used to describe the status of the samples in each group using frequency distribution tables and summary statistics, such as mean and standard deviation. At the inferential level, a Shapiro‐Wilk test was conducted to test the normality assumption for the data distribution. Analyses based on parametric tests are appropriate if the data meet the normality assumption. If the assumption of normality is not met, non‐parametric tests are used.

To compare multiple groups, an analysis of variance (ANOVA) was conducted following confirmation of normality. Inferences were drawn about the differences between the three groups based on these tests. A significance level of 5% was applied to all analyses, and SPSS version 24 was used for the analysis. On the basis of these statistical tests, comparisons between groups were made, and conclusions were reached.

## 3 RESULTS

When using Bone^+^B® graft in the bone defect in the rabbit calvarium, the percentage of bone formation in the range of 32% to 45% was observed with a mean (±standard deviation) of 37.80% (±5.63), with a 95% confidence interval of 30.81% to 44.79%. The percentage of remaining material ranged from 28% to 37%, with a mean (±standard deviation) of 32.60% (±3.65), with a 95% confidence interval of 28.07% to 37.13%.

When using InterOss® graft in the bone defect in the rabbit calvarium, the percentage of bone formation in the range of 61% to 75% was observed with a mean (±standard deviation) of 65.83% (±4.75), with a 95% confidence interval of 60.85% to 70.82%. The percentage of remaining material ranged from 9% to 18%, with a mean (±standard deviation) of 13.17% (±3.06), with a 95% confidence interval of 9.95% to 16.38%.

There was no residual material in the control group. There was a range in percentage of bone formation among this group, ranging from 10% to 25%, with a mean (±standard deviation) of 17.17% (±6.11). For the control group, the 95% confidence interval ranged from 10.75% to 23.58%.

The performance of Bone^+^B® grafts and InterOss® grafts in rabbit calvarium defects was distinguished by notable differences across key parameters. Bone^+^B® showed a lower mean percentage of bone formation than InterOss®, indicating its superior osteogenic potential. Additionally, InterOss® had a lower percentage of remaining material, suggesting a quicker graft resorption.

Statistical analysis of new bone formation among the groups revealed a significant increase in both groups Bone^+^B® and InterOss® compared to the control group (*p* < .05). Also, in comparing two xenografts with each other, the level of bone formation was higher in the InterOss® group compared to Bone^+^B® (*p* < .05). Regarding the amount of remaining graft at the site, it was significantly lower in the InterOss® group compared to Bone^+^B® (*p* < .05), indicating potentially higher absorption of InterOss®.

According to the results of this study, inflammation levels in the InterOss® group were lower than in the Bone^+^B® group, although the difference did not reach statistical significance (*p* > .05). Therefore, despite a difference in inflammation levels between the two groups, these differences were not significant enough to conclude which graft material caused less inflammation (Table 1).

**Table 1:** The comparison of the rate of new bone formation, the amount of remaining material from the graft, and the level of inflammation in three groups of rabbits after three months of Initiation of defects based on histopathological analysis.

Bone formation								
	Number	Mean	Minimum	Maximum	Standard deviation	Confidence interval	Lower bound	Upper bound
Bone^+^B®	5	37.80%	32%	45%	5.63	95%	30.81%	44.79%
InterOss®	6	65.83%	61%	75%	4.75	95%	60.85%	70.82%
Control	6	17.17%	10%	25%	17.17	95%	10.75%	23.58%
*p* value	*p* < .05							

Remaining material								
	Number	Mean	Minimum	Maximum	Standard deviation	Confidence interval	Lower bound	Upper bound
Bone^+^B®	5	32.60%	28%	37%	3.65	95%	28.07%	37.13%
InterOss®	6	13.17%	9%	18%	3.06	95%	9.95%	16.38%
*p* value	*p* < .05							

Inflammation level								
	Number	Without inflammation (grade 0)		Minor inflammation (grade 1)		*p* value		
Bone^+^B®	5	4	80%	1	20%	*p* > .05		
InterOss®	6	5	83.33%	1	16.66%			
Control	6	5	83.33%	1	16.66%

## 4 DISCUSSION

Based on the findings of the present study, InterOss® Xenograft performed better than Bone^+^B® in terms of bone formation and remaining material after implanting. The two products, InterOss® and Bone^+^B®, are both made from bovine cancellous bone. Results may differ based on the method of processing each xenograft, based on chemical or physical procedures. During InterOss® processing, reduced temperatures and low heating rates may have contributed to better results (Valencia‐Llano et al., 2022a). Bone^+^B® powder's performance may have been compromised by the retention of bovine immunogenic materials following suboptimal processing.

When it comes to the production of xenografts, Mammalian bones can be utilized, as well as fish bony and scale materials, bird eggshells, and exoskeletons of marine organisms (Suh et al., 2001). In terms of their chemical composition and structure, they resemble human bone tissue, so they tend to be biodegradable and physiologically compatible (Dumitrescu et al., 2021; Kao & Scott, 2007).

Currently, deproteinized cancellous bovine bone matrix (DBBM) is most commonly used for the reconstruction of alveolar bone defects and the augmentation of sinus floors (Jensen et al., 2012; Pang et al., 2017). One of the most commonly used DBBMs in dentistry is Bio‐Oss®. It is highly biocompatible with hard tissues, has a porous texture similar to human bone, and has good bone conductivity (Berglundh & Lindhe, 1997; Haas et al., 1998; Lee et al., 2017; PINHOLT et al., 1991).

An investigation has shown that DBBM and collagen matrix are effective in sealing non‐molar excision sites with variable degrees of alveolar ridge atrophy, primarily in the facial region. Despite a number of reports of alveolar ridge atrophy, alveolar ridge preservation (ARP) therapy is successful (Couso‐Queiruga et al., 2023).

In a study by Bae et al., porcine‐derived xenografts (Bone‐XP®) were compared with bovine‐derived xenografts (Bio‐Oss®) for use in rat calvarial defects when compared to the porcine‐derived xenografts (Bone‐XP®). Resulting analyses showed that human mesenchymal stem cells differentiate osteogenically without toxicity, similar to the results of the present study. Therefore, they concluded that porcine‐derived bone substitute, Bone‐XP®, may provide similar cell responses and bone regeneration as commercial bovine bone mineral (Bae et al., 2019).

Park et al. (2023) integrated a novel guided bone regeneration (GBR) strategy by applying rapid bone‐forming growth factors (GF) to the membrane outside the bone defect, aiming to enhance osteogenic ability (Park et al., 2023). Using a dual scaffold complex in conjunction with collagen membrane and biphasic calcium phosphate (BCP), four 8 mm bone defects in New Zealand white rabbits were treated with varying concentrations of BMP‐2 or FGF‐2. The experimental groups exhibited continuous forms of new bones in the upper part of the bone defect, as opposed to the control group, in histological analysis. Notably, histomorphometric results showed a significant increase in new bone formation with the application of BMP‐2 (0.5 mg/mL) and FGF‐2 (1.0 mg/mL) and a time‐dependent effect with higher new bone formation at 8 weeks compared to 2 and 4 weeks. In conclusion, this integrated GBR method, incorporating specific growth factor concentrations and a dual scaffold complex, proves effective for bone regeneration, showcasing both quantitative and qualitative advantages for sustained bone maintenance over time.

Shiu et al. (2021) assessed the new bone formation potential of micro‐macro biphasic calcium phosphate (MBCP) and Bio‐Oss® grafting materials with and without dental pulp‐derived mesenchymal stem cells (DPSCs) and bone marrow‐derived mesenchymal stem cells (BMSCs) in a rabbit calvaria bone defect model (Shiu et al., 2021). The grafting materials exhibited homogeneously porous structures, and both DPSCs and BMSCs enhanced osteoconductive capacities. Autogenous bone demonstrated the highest new bone formation, while MBCP and Bio‐Oss® supported osteoconduction and prevented defect space collapse. Incorporating DPSCs and BMSCs resulted in increased osteoblastic cells lining the newly formed bone and grafting materials. Notably, the combination of BMSCs with MBCP and Bio‐Oss® showed comparable efficiency to autogenous bone after 8 weeks, providing effective strategies for improving biomaterials and mesenchymal stem cell‐based bone tissue regeneration.

Lee et al. (2014) reported significant similarities in pore structure, microstructure, structure phase, and chemical composition in InterOss® bone powder, which had been commercially available since 1995 as an alternative to Bio‐Oss®. According to BET analysis, InterOss® has a relatively higher internal surface area than Bio‐Oss®. In particular, protein analysis showed that InterOss® possessed a relatively lower residual protein content than Bio‐Oss®. InterOss® may prove to be a promising bone graft material in periodontics and maxillofacial surgery due to its similarities in physical and chemical characteristics, increased surface area, and low residual protein content (Lee et al., 2014). These results align with the current study, which demonstrated that InterOss® grafts regenerate bone tissue more effectively. Their compatibility was also demonstrated by significant reductions in remnant material content.

Researchers studied the biological behavior of three materials that can be applied to bone tissue engineering via in vitro experiments and in vivo implantations. InterOss®, an inorganic bovine apatite particulate, was among the samples. Due to its long‐term stability, high porosity, and resorbability, this mineralized osteoconductive structure served well as a substitute for human bone (Valencia‐Llano et al., 2022a).

Using a rabbit calvaria model, synthetic bone substitutes were compared to bio‐osseous xenografts (Bio‐Oss®).  Histomorphometric and micro‐CT analysis was performed by Tovar et al. (2014) at 4 and 8‐weeks following implant placement to evaluate the new bone formation. A similar level of bone formation was achieved by both methods, while the control group showed a lower level of soft tissue infiltration and bone formation. Experimental materials showed greater remodeling activity and lower graft degradation compared to commercial xenografts at 8 weeks, but there were no significant differences in quantitative parameters (Tovar et al., 2014).

The infiltration of dense polymorphonuclear cells and acute inflammation are common findings in studies involving synthetic or xenograft materials. Slowly absorbed materials, such as hydroxyapatite, can cause chronic inflammation and even be encapsulated by fibrous tissue (Mariani et al., 2019). Due to the appropriate structure and quality of the grafts used in this study, inflammation levels were mild overall due to the possibility of bone remodeling being impaired by acute inflammation. Reaffirming the quality of the grafts used in this case is this observation. All groups in this study displayed mild inflammation, suggesting high biocompatibility and minimal immune response to the graft materials, which is consistent with previous studies (Paknejad et al., 2015; Rokn et al., 2015).

## 5 CONCLUSION

According to the results of this study, InterOss® bone powder offered better bone reconstruction, less residual material, and lower inflammation level in comparison with Bone^+^B® during the 3‐month healing period. Consequently, InterOss® bone powder should be the superior choice for maxillofacial and facial surgery. However, since the Bone^+^B® powder group showed significant improvements in healing compared with the control group, primarily due to better bone formation, Bone^+^B® bone powder may also be prescribed in certain circumstances.

## AUTHOR CONTRIBUTIONS

*Conceptualization*: Sepehr Kobravi. *Methodology*: All authors. *Formal analysis and investigation*: All authors. *Writing–original draft preparation*: Narges Lotfalizadeh. *Writing–review and editing*: All authors. *Funding acquisition*: No fund. *Supervision*: Afshin Yadegari Nain. All authors checked and approved the final version of the manuscript for publication in the present journal.

## CONFLICT OF INTEREST STATEMENT

The authors declare no conflict of interest.

## ETHICS STATEMENT

All applicable international, national, and/or institutional guidelines for the care and use of animals were followed. Ethical code: IR. IAU. KHUISF. REC.1400.165.

## Supporting information

Supporting information.

## Data Availability

The datasets generated during and/or analyzed during the current study are available from the corresponding author upon reasonable request.

## References

[cre2875-bib-0001] Bae, E.‐B. , Kim, H.‐J. , Ahn, J.‐J. , Bae, H.‐Y. , Kim, H.‐J. , & Huh, J.‐B. (2019). Comparison of bone regeneration between porcine‐derived and bovine‐derived xenografts in rat calvarial defects: A non‐inferiority study. Materials, 12(20), 3412. 31635277 10.3390/ma12203412 PMC6829332

[cre2875-bib-0002] Berglundh, T. , & Lindhe, J. (1997). Healing around implants placed in bone defects treated with Bio‐Oss®. An experimental study in the dog. Clinical Oral Implants Research, 8(2), 117–124. 9758962 10.1034/j.1600-0501.1997.080206.x

[cre2875-bib-0003] Caccamese, J. F. (2023). Donor site options, Cleft maxillary reconstruction (pp. 77–88). Springer.

[cre2875-bib-0004] Canellas, J. V. D. S. , Drugos, L. , Ritto, F. G. , Fischer, R. G. , & Medeiros, P. J. D. (2021). Xenograft materials in maxillary sinus floor elevation surgery: A systematic review with network meta‐analyses. British Journal of Oral and Maxillofacial Surgery, 59(7), 742–751. 34120778 10.1016/j.bjoms.2021.02.009

[cre2875-bib-0005] Capella‐Monsonís, H. , & Zeugolis, D. I. (2021). Decellularized xenografts in regenerative medicine: From processing to clinical application. Xenotransplantation, 28(4), e12683. 33709410 10.1111/xen.12683

[cre2875-bib-0006] Chang, Y.‐Y. , Dissanayake, S. , Yun, J.‐H. , Jung, U.‐W. , Kim, C.‐S. , Park, K.‐J. , Chai, J. K. , & Choi, S. H. (2011). The biological effect of cyanoacrylate‐combined calcium phosphate in rabbit calvarial defects. Journal of Periodontal & Implant Science, 41(3), 123–130. 21811687 10.5051/jpis.2011.41.3.123 PMC3139045

[cre2875-bib-0007] Couso‐Queiruga, E. , Weber, H. A. , Garaicoa‐Pazmino, C. , Barwacz, C. , Kalleme, M. , Galindo‐Moreno, P. , & Avila‐Ortiz, G. (2023). Influence of healing time on the outcomes of alveolar ridge preservation using a collagenated bovine bone xenograft: A randomized clinical trial. Journal of Clinical Periodontology, 50(2), 132–146. 36345818 10.1111/jcpe.13744 PMC10100450

[cre2875-bib-0008] Dumitrescu, C. R. , Neacsu, I. A. , Surdu, V. A. , Nicoara, A. I. , Iordache, F. , Trusca, R. , Ciocan, L. T. , Ficai, A. , & Andronescu, E. (2021). Nano‐hydroxyapatite vs. xenografts: Synthesis, characterization, and in vitro behavior. Nanomaterials, 11(9), 2289. 34578603 10.3390/nano11092289 PMC8469747

[cre2875-bib-0009] Fishman, J. A. (2022). Risks of infectious disease in xenotransplantation. New England Journal of Medicine, 387(24), 2258–2267. 36516091 10.1056/NEJMra2207462

[cre2875-bib-0010] Gashtasbi, F. , Hasannia, S. , Hasannia, S. , Mahdi Dehghan, M. , Sarkarat, F. , & Shali, A. (2020). Comparative study of impact of animal source on physical, structural, and biological properties of bone xenograft. Xenotransplantation, 27(6), e12628. 32654298 10.1111/xen.12628

[cre2875-bib-0011] Haas, R. , Donath, K. , Födinger, M. , & Watzek, G. (1998). Bovine hydroxyapatite for maxillary sinus grafting: Comparative histomorphometric findings in sheep. Clinical Oral Implants Research, 9(2), 107–116. 9663038 10.1034/j.1600-0501.1998.090206.x

[cre2875-bib-0012] Hussain, I. , Moharamzadeh, K. , Brook, I. M. , José de Oliveira Neto, P. , & Salata, L. A. (2012). Evaluation of osteoconductive and osteogenic potential of a dentin‐based bone substitute using a calvarial defect model. International Journal of Dentistry, 2012, 1–7. 10.1155/2012/396316 PMC3312261 22505899

[cre2875-bib-0013] Imaniyyah, A. G. , & Herda, E. (2022). Monetite as a potential ideal bone substitute: A short review on fabrication and properties. Materials Today: Proceedings, 66, 2762–2766.

[cre2875-bib-0014] Jensen, T. , Schou, S. , Stavropoulos, A. , Terheyden, H. , & Holmstrup, P. (2012). Maxillary sinus floor augmentation with bio‐oss or bio‐oss mixed with autogenous bone as graft: A systematic review. Clinical Oral Implants Research, 23(3), 263–273. 21443592 10.1111/j.1600-0501.2011.02168.x

[cre2875-bib-0015] Josephson, C. D. , & Kuehnert, M. J. (2022). Human tissue allografts: Responsibilities in understanding the path from donor to recipient. Rossi's Principles of Transfusion Medicine, 58, 660–673.

[cre2875-bib-0016] Kao, S. T. , & Scott, D. D. (2007). A review of bone substitutes. Oral and maxillofacial surgery clinics of North America, 19(4), 513–521. 18088902 10.1016/j.coms.2007.06.002

[cre2875-bib-0017] Lee, D. S. H. , Pai, Y. , & Chang, S. (2014). Physicochemical characterization of InterOss and Bio‐Oss anorganic bovine bone grafting material for oral surgery–A comparative study. Materials Chemistry and Physics, 146(1–2), 99–104.

[cre2875-bib-0018] Lee, E.‐H. , Kim, J.‐Y. , Kweon, H. Y. , Jo, Y.‐Y. , Min, S.‐K. , Park, Y.‐W. , Choi, J. Y. , & Kim, S. G. (2010). A combination graft of low‐molecular‐weight silk fibroin with Choukroun platelet‐rich fibrin for rabbit calvarial defect. Oral Surgery, Oral Medicine, Oral Pathology, Oral Radiology, and Endodontology, 109(5), e33–e38. 10.1016/j.tripleo.2009.12.043 20149696

[cre2875-bib-0019] Lee, J. H. , Yi, G. S. , Lee, J. W. , & Kim, D. J. (2017). Physicochemical characterization of porcine bone‐derived grafting material and comparison with bovine xenografts for dental applications. Journal of Periodontal & Implant Science, 47(6), 388–401. 29333325 10.5051/jpis.2017.47.6.388 PMC5764765

[cre2875-bib-0020] Mariani, E. , Lisignoli, G. , Borzì, R. M. , & Pulsatelli, L. (2019). Biomaterials: Foreign bodies or tuners for the immune response? International Journal of Molecular Sciences, 20(3), 636. 30717232 10.3390/ijms20030636 PMC6386828

[cre2875-bib-0021] Monje, A. , & Nart, J. (2022). Management and sequelae of dental implant removal. Periodontology 2000, 88(1), 182–200. 35103326 10.1111/prd.12418

[cre2875-bib-0022] Öztürk, K. , Kahraman, S. , & Delilbaşı, E. (2021). Evaluation of early bone recovery in grafted jaw with anterior iliac bone: A retrospective study. Journal of Osseointegration, 13(3), 109–114.

[cre2875-bib-0023] Paknejad, M. , Rokn, A. , Rouzmeh, N. , Heidari, M. , Titidej, A. , Kharazifard, M. J. , & Mehrfard, A. (2015). Histologic evaluation of bone healing capacity following application of inorganic bovine bone and a new allograft material in rabbit calvaria. Journal of Dentistry (Tehran, Iran), 12(1), 31–38. 26005452 PMC4436325

[cre2875-bib-0024] Pang, K. M. , Um, I. W. , Kim, Y. K. , Woo, J. M. , Kim, S. M. , & Lee, J. H. (2017). Autogenous demineralized dentin matrix from extracted tooth for the augmentation of alveolar bone defect: A prospective randomized clinical trial in comparison with anorganic bovine bone. Clinical Oral Implants Research, 28(7), 809–815. 27279547 10.1111/clr.12885

[cre2875-bib-0025] Park, J. , Jung, N. , Lee, D.‐J. , Oh, S. , Kim, S. , Cho, S.‐W. , Kim, J. E. , Moon, H. S. , & Park, Y. B. (2023). Enhanced bone formation by rapidly formed bony wall over the bone defect using dual growth factors. Tissue Engineering and Regenerative Medicine, 20, 767–778. 37079199 10.1007/s13770-023-00534-z PMC10352230

[cre2875-bib-0026] Pinholt, E. M. , Bang, G. , & Haanaes, H. R. (1991). Alveolar ridge augmentation in rats by Bio‐Oss. European Journal of Oral Sciences, 99(2), 154–161. 10.1111/j.1600-0722.1991.tb01878.x 1828906

[cre2875-bib-0027] Raghoebar, G. M. , Timmenga, N. M. , Reintsema, H. , Stegenga, B. , & Vissink, A. (2001). Maxillary bone grafting for insertion of endosseous implants: results after 12–124 months. Clinical Oral Implants Research, 12(3), 279–286. 11359486 10.1034/j.1600-0501.2001.012003279.x

[cre2875-bib-0028] Robinson, P. G. , Abrams, G. D. , Sherman, S. L. , Safran, M. R. , & Murray, I. R. (2020). Autologous bone grafting. Operative Techniques in Sports Medicine, 28(4), 150780.

[cre2875-bib-0029] Rokn, A. R. , Shakeri, A. S. , Etemad‐Moghadam, S. , Alaeddini, M. , Shamshiri, A. R. , Manasheof, R. , & Barikani, H. (2015). Regenerative effects of three types of allografts on rabbit calvarium: An animal study. Journal of Dentistry (Tehran, Iran), 12(11), 823–834. 27507993 PMC4977406

[cre2875-bib-0030] Sakkas, A. , Wilde, F. , Heufelder, M. , Winter, K. , & Schramm, A. (2017). Autogenous bone grafts in oral implantology—is it still a “gold standard”? A consecutive review of 279 patients with 456 clinical procedures. International Journal of Implant Dentistry, 3, 23. 28573552 10.1186/s40729-017-0084-4 PMC5453915

[cre2875-bib-0031] Schlund, M. , Depeyre, A. , Kotagudda Ranganath, S. , Marchandise, P. , Ferri, J. , & Chai, F. (2022). Rabbit calvarial and mandibular critical‐sized bone defects as an experimental model for the evaluation of craniofacial bone tissue regeneration. Journal of Stomatology, Oral and Maxillofacial Surgery, 123(6), 601–609. 34902627 10.1016/j.jormas.2021.12.001

[cre2875-bib-0032] Sheikh, Z. , Hamdan, N. , Ikeda, Y. , Grynpas, M. , Ganss, B. , & Glogauer, M. (2017). Natural graft tissues and synthetic biomaterials for periodontal and alveolar bone reconstructive applications: A review. Biomaterials Research, 21(1), 9. 28593053 10.1186/s40824-017-0095-5 PMC5460509

[cre2875-bib-0033] Shiu, S.‐T. , Lee, W.‐F. , Chen, S.‐M. , Hao, L.‐T. , Hung, Y.‐T. , Lai, P.‐C. , & Feng, S.‐W. (2021). Effect of different bone grafting materials and mesenchymal stem cells on bone regeneration: A micro‐computed tomography and histomorphometric study in a rabbit calvarial defect model. International Journal of Molecular Sciences, 22(15), 8101. 34360864 10.3390/ijms22158101 PMC8347101

[cre2875-bib-0034] Suh, H. , Han, D. W. , Park, J. C. , Lee, D. H. , Lee, W. S. , & Han, C. D. (2001). A bone replaceable artificial bone substitute: Osteoinduction by combining with bone inducing agent. Artificial Organs, 25(6), 459–466. 11453876 10.1046/j.1525-1594.2001.025006459.x

[cre2875-bib-0035] Titsinides, S. , Agrogiannis, G. , & Karatzas, T. (2019). Bone grafting materials in dentoalveolar reconstruction: A comprehensive review. Japanese dental science review, 55(1), 26–32. 30733842 10.1016/j.jdsr.2018.09.003 PMC6354279

[cre2875-bib-0036] Tovar, N. , Jimbo, R. , Gangolli, R. , Perez, L. , Manne, L. , Yoo, D. , Lorenzoni, F. , Witek, L. , & Coelho, P. G. (2014). Evaluation of bone response to various anorganic bovine bone xenografts: An experimental calvaria defect study. International Journal of Oral and Maxillofacial Surgery, 43(2), 251–260. 23948358 10.1016/j.ijom.2013.07.005

[cre2875-bib-0037] Valencia‐Llano, C. H. , López‐Tenorio, D. , & Grande‐Tovar, C. D. (2022a). Biocompatibility assessment of two commercial bone xenografts by in vitro and in vivo methods. Polymers, 14(13), 2672. 35808724 10.3390/polym14132672 PMC9268806

[cre2875-bib-0038] Valencia‐Llano, C. H. , López‐Tenorio, D. , Saavedra, M. , Zapata, P. A. , & Grande‐Tovar, C. D. (2022b). Comparison of two bovine commercial xenografts in the regeneration of critical cranial defects. Molecules, 27(18), 5745. 36144483 10.3390/molecules27185745 PMC9506155

[cre2875-bib-0039] Zhang, J. , Li, S. , He, H. , Han, L. , Zhang, S. , Yang, L. , Han, W. , Wang, X. , Gao, J. , Zhao, J. , & Shi, W. (2023). Clinical guidelines for indications, techniques, and complications of autogenous bone grafting. Chinese Medical Journal, 10, 1097. 10.1097/CM9.0000000000002691 PMC10766296 37462050

